# Ocular biometrics and uncorrected visual acuity for detecting myopia in Chinese school students

**DOI:** 10.1038/s41598-022-23409-0

**Published:** 2022-11-04

**Authors:** Ethan Zhao, Xinyi Wang, Huiyan Zhang, Eric Zhao, Jianyong Wang, Ying Yang, Fang Gu, Lei Gu, Jianyao Huang, Ronghua Zhang, Gui-shuang Ying, Hongguang Cui

**Affiliations:** 1 Weill Cornell Medicine, New York, NY USA; 2 National School of Development, Peking University, Beijing, People’s Republic of China; 3 Hangzhou Vocational and Technical College, Hangzhou, People’s Republic of China; 4 School of Engineering and Applied Science, University of Pennsylvania, Philadelphia, PA USA; 5 Department of Ophthalmology, The First Affiliated Hospital, College of Medicine, Zhejiang University, Hangzhou, People’s Republic of China; 6 Center for Disease Control and Prevention of Jinyun County, Jinyun, Zhejiang People’s Republic of China; 7 Zhejiang Provincial Center for Disease Control and Prevention of Hangzhou, Hangzhou, People’s Republic of China; 8 Department of Ophthalmology, Central Hospital of Jinyun County, Jinyun, Zhejiang People’s Republic of China; 9 Center for Preventive Ophthalmology and Biostatistics, Department of Ophthalmology, Perelman School of Medicine, University of Pennsylvania, 3711 Market Street, Suite 801, Philadelphia, PA 19104 USA

**Keywords:** Refractive errors, Vision disorders

## Abstract

The study is to evaluate the performance of ocular biometric measures and uncorrected visual acuity (UCVA) for detecting myopia among Chinese students. Among 5- to 18-year-old Chinese students from two cities of China, trained eye-care professionals performed assessment of ocular biometrics (axial length (AL), corneal curvature radius (CR), anterior chamber depth) under noncycloplegic conditions using NIDEK Optical Biometer AL-Scan, distance visual acuity using retro-illuminated logMAR chart with tumbling-E optotypes, and cycloplegic refractive error using NIDEK autorefractor with administration of 0.5% tropicamide. Spherical equivalent (SER) in diopters (D) was calculated as sphere plus half cylinder, and myopia was defined as SER ≤ − 0.5 D. Performances of ocular biometrics and UCVA (individually and in combination) for detecting myopia were evaluated using sensitivity and specificity, predictive values, and area under ROC curve (AUC) in both development dataset and validation dataset. Among 3436 students (mean age 9.7 years, 51% female), the mean (SD) cycloplegic SER was − 0.20 (2.18) D, and 1269 (36.9%) had myopia. Cycloplegic SER was significantly correlated with AL (Pearson Correlation coefficient r = − 0.82), AL/CR ratio (r = − 0.90), and UCVA (r = 0.79), but was not correlated with CR (r = 0.02, p = 0.15). The AL/CR ratio detected myopia with AUC 0.963 (95% CI 0.957–0.969) and combination with UCVA improved the AUC to 0.976 (95% CI 0.971–0.981). Using age-specific AL/CR cutoff (> 3.00 for age < 10 years, > 3.06 for 10–14 years, > 3.08 for ≥ 15 years) as myopia positive, the sensitivity and specificity were 87.0% (95% CI 84.4–89.6%) and 87.8% (86.0–89.6%), respectively, in the development dataset and 86.4% (95% CI 83.7–89.1%) and 89.4% (95% CI 87.3–91.4%), respectively, in the validation dataset. Combining AL/CR and UCVA (worse than 20/32 for age < 10 years, and 20/25 for ≥ 10 years) provided 91.9% (95% CI 90.4–93.4%) sensitivity and 87.0% (95% CI 85.6–88.4%) specificity, positive value of 80.6% (95% CI 78.5–82.6%) and negative value of 94.8% (95% CI 93.8–95.8%). These results suggest that AL/CR ratio is highly correlated with cycloplegic refractive error and detects myopia with high sensitivity and specificity,  AL/CR ratio alone or in combination with UCVA can be used as a tool for myopia screening or for estimating myopia prevalence in large epidemiological studies with limited resources for cycloplegic refraction.

## Introduction

Myopia is a major public health problem worldwide, particularly in East and Southeast Asia where myopia is already at the epidemic level^1^. The prevalence of myopia is increasing throughout the world, and the number of individuals with myopia is predicted to increase to 4.8 billion by 2050^1^. The risk of myopia increases dramatically from approximately 6 years of age until age 18 years old, with myopia rates as high as 80% for the urban Han population in China^2^. Because myopia is associated with many ophthalmic diseases, including retinal detachment, glaucoma and maculopathy^3^, and there are effective interventions available for myopia control^4^, identifying children with myopia or at high risk of developing myopia for timely intervention are crucial from a public health perspective.

Cycloplegic refractive error is the gold standard for the detection of myopia^5^. However, it sometimes can be challenging to administer cycloplegic eyedrops to children, particularly in large-scale studies with limited resources for cycloplegic refraction. Thus, noncycloplegic refractive error is still commonly used for determining the presence or severity of myopia in some population-based epidemiological studies of pediatric myopia^6–8^, even though noncycloplegic refractive error is well-known to overestimate myopia prevalence and severity.

Many previous studies reported the high correlation of ocular biometric measures with the refractive error^9–15^, with highest correlation between AL/CR ratio and cycloplegic spherical equivalent ranging from − 0.78 to − 0.89 in young adults and from − 0.61 to − 0.78 in school children^10–16^. Motivated by the high correlation between of biometric measures and refractive error, previous studies have assessed the accuracy of detecting or predicting the myopia using ocular biometric measures individually^17^, or in combination with noncycloplegic refractive error, and uncorrected visual acuity^18^. These studies yielded mixed results, with various sensitivity/specificity, likely due to variations in sample size, children’s ages, their refractive error status, and cutoff values of measures for defining myopia-positive^9–12,17–19^. Large studies are needed to further evaluate their performance before they can be applied to screen for myopia or to determine myopia prevalence in large epidemiological studies.

In this large cross-sectional school-based myopia study of Chinese school students aged 5–18 years from two cities in China, we aim to evaluate the performance of using ocular biometric measures (e.g., axial length, corneal curvature radius, anterior chamber depth, etc.) and uncorrected visual acuity (UCVA) for detecting myopia. We hypothesized that using optimal cutoff values derived in this study and validated independently, certain ocular biometrics alone or in combination with UCVA is able to detect the myopia with high sensitivity and specificity, thus potentially providing a valid tool for screening myopia or estimating myopia prevalence in large myopia studies with limited resources for cycloplegic refraction.

## Methods

The details on the study design and measures of refractive error were already described in previous publications^20,21^. Only the information on measures of ocular biometrics, uncorrected visual acuity and cycloplegic refractive error related to this paper were described here.

This cross-sectional school-based study of myopia was conducted October 2020 to January 2021 in two cities (Jinyun, Hangzhou) of the central Zhejiang province, People’s Republic of China. In each city, three kindergartens, one elementary school (grade 1 to 6), one middle school (grade 7–9) and one high school (grade 10–12) were randomly selected. Among the selected schools, a random sample of classes from each grade were selected and all students from the selected classes were invited to participate the study.

The participating students between 5 and 18 years of age from Jinyun (N = 1938) and Hangzhou (N = 1498) underwent comprehensive ocular assessments by trained eye-care professionals (optometrists or ophthalmologists) following the standard study protocol. Each student was tested by a team of three trained optometrists or ophthalmologists for ocular measures following the order of: (1) distance visual acuity using retro-illuminated logMAR chart with tumbling-E optotypes; (2) ocular biometrics including axial length (AL), corneal curvature radius (CR), central corneal thickness (CCT), anterior chamber depth (ACD) under noncycloplegic condition using Optical Biometer AL-Scan (NIDEK, Japan); and (3) noncycloplegic and cycloplegic refractive error using table-mounted NIDEK autorefractor (Model: ARK-510A, Japan). For cycloplegic refractive error, one drop of commercial 0.5% tropicamide was instilled in each eye. A second, third and fourth drop of 0.5% tropicamide was instilled in each eye every 5 min. Thirty minutes after the fourth drop of 0.5% tropicamide was instilled, three readings of refractive error (sphere, cylinder and axis) were taken from each eye. If the difference between any two readings from an eye was greater than 0.5 diopters (D), the refractive error for that eye was re-taken. The average of the three readings of refractive error of each eye was entered into a database for statistical analysis.

For the test of visual acuity, students were asked whether they wear glasses, contact lens or orthokeratology contact lens. For the students not wearing glasses, the uncorrected visual acuity (UCVA) was tested for each eye. For those who wear glasses, visual acuity was first measured without correction, then was measured with best correction at 30 min after removing the glasses.

The study followed the tenets of the Declaration of Helsinki. Human subject research approval was obtained from Zhejiang University and the local Administration of the Education and School Board. Written informed consent was obtained from legal parents/guardians. In addition, for students with age 18 years or older, the assent from students was also obtained.

### Statistical analyses

Cycloplegic spherical equivalent (SER) was calculated as sphere plus half of the cylinder for each eye. Myopia was defined as cycloplegic SER − 0.5 D or worse in either eye, and high myopia was defined as cycloplegic SER − 6.0 D or worse in either eye. We performed the Pearson correlation analysis between cycloplegic SER and each of ocular biometric measures including AL, CR, AL/CR ratio, CCT, ACD, and UCVA, and compared biometric measures between students with vs. without myopia. In these analyses, the average of two eyes of each student was used because of the high inter-eye correlation in these measures (inter-eye correlation coefficient > 0.92 for each biometric measure and refractive error).

To evaluate the performance of each ocular biometric measure and UCVA for detecting myopia, we calculated the sensitivity and specificity, positive predictive value (PPV) and negative predictive value (NPV), the area under ROC curve (AUC) and their 95% confidence intervals (95% CI). We evaluated the performance of these ocular biometric measures individually and their combinations with UCVA to determine whether inclusion of UCVA improves the performance of ocular biometric measures for detecting myopia.

To identify the optimal cutoff value for each ocular biometric measure and UCVA, we calculated the sensitivity and specificity corresponding to each possible cutoff values for each year age in the development dataset from students of Jinyun (N = 1938). However, because some age groups had small number of students or small number of myopia cases, we combined students into 3 age groups (< 10, 10–14 and ≥ 15 years) based on their similarity of the AL/CR distribution. Because it is desirable to have high sensitivity (> 80%) for detecting myopia for each age group, the age-specific optimal cutoff values were determined primarily based on the high sensitivity and high Youden index calculated as the sum of sensitivity and specificity minus one^22^. The determined age-specific optimal cutoff values were then independently validated in the validation dataset from the students of Hangzhou (N = 1498). The overall performance of the optimal cutoff points was assessed in a combined dataset containing all study subjects (N = 3436). We calculated the sensitivity and specificity of using AL, AL/CR ratio and UCVA separately for detecting myopia. In addition, the sensitivity and specificity from the combination of AL/CR ratio and UCVA were calculated, because the AUC from this combination is much higher than that from each individual biometric measure and the combination of AL and UCVA. Given the high inter-eye correlation of biometrics and UCVA, we calculated the sensitivity, specificity and predictive values at person level, i.e., a student was defined as myopia-positive if their ocular biometrics or UCVA measures in either eye were worse than the determined cutoff values. All statistical analyses were performed in SAS v9.4 (SAS Institute Inc, Cary, NC, USA) and two-sided p < 0.05 (without correction for multiple comparison) was considered statistically significant.

## Results

### Characteristics of study participants

The study included 3436 school-aged students (1938 students from Jinyun for cutoff value determination and 1498 students from Hangzhou for performance validation using selected cutoff points). Student characteristics are shown in Table 1. Among all 3436 children, 1740 (50.6%) were females and 1696 (49.4%) males, and their age ranged from 5 to 18 years, with 613 (17.8%) kindergarteners, 2025 (59.0%) elementary school students, 424 (12.3%) middle schoolers, and 374 (10.9%) high schoolers. The mean (SD) cycloplegic SER was − 0.20 (2.18) D, with 1269 (36.9%) having myopia and 91 (2.7%) having high myopia in one or both eyes. UCVA 20/200 or worse was observed in 160 (4.7%) students, and 20/20 or better in 1956 (56.9%) students. The means (SD) of biometric measures were 23.6 (1.3) mm for axial length, 7.84 (0.26) mm for corneal curvature radius, 3.02 (0.15) for AL/CR ratio, and 3.60 (0.32) mm for anterior chamber depth.

**Table 1:** Characteristics of school students (N = 3436).

Characteristics of children	Development dataset: Jinyun (N = 1938)	Validation dataset: Hangzhou (N = 1498)	All (N = 3436)
**Age (years), n (%)**			
5	35 (1.8%)	23 (1.5%)	58 (1.7%)
6	429 (22.1%)	227 (15.2%)	656 (19.1%)
7	317 (16.4%)	269 (18.0%)	586 (17.1%)
8	210 (10.8%)	218 (14.6%)	428 (12.5%)
9	148 (7.6%)	151 (10.1%)	299 (8.7%)
10	132 (6.8%)	70 (4.7%)	202 (5.9%)
11	148 (7.6%)	91 (6.1%)	239 (7.0%)
12	109 (5.6%)	82 (5.5%)	191 (5.6%)
13	82 (4.2%)	83 (5.5%)	165 (4.8%)
14	69 (3.6%)	57 (3.8%)	126 (3.7%)
15	65 (3.4%)	56 (3.7%)	121 (3.5%)
16	72 (3.7%)	59 (3.9%)	131 (3.8%)
17	61 (3.2%)	58 (3.9%)	119 (3.5%)
18	61 (3.2%)	54 (3.6%)	115 (3.4%)
Mean (SD)	9.6 (3.6)	9.9 (3.6)	9.7 (3.6)
Gender: female (%)	999 (51.2%)	741 (49.5%)	1740 (50.6%)
**Grade, n (%)**			
Kindergarten	414 (21.4%)	199 (13.3%)	613 (17.8%)
Elementary schooler	1105 (57.0%)	920 (61.4%)	2025 (58.9%)
Middle schooler	223 (11.5%)	201 (13.4%)	424 (12.3%)
High schooler	196 (10.1%)	178 (11.9%)	374 (10.9%)
Cycloplegic SER in most myopic eye (diopters): mean (SD)	− 0.07 (2.10)	− 0.37 (2.26)	− 0.20 (2.18)
Myopia in either eye: yes (%)*	652 (33.6%)	617 (41.2%)	1269 (36.9%)
High myopia in either eye: Yes (%)**	40 (2.1%)	51 (3.4%)	91 (2.7%)
**Uncorrected visual acuity in worse eye: n (%)**			
20/200 or worse	62 (3.2%)	98 (6.5%)	160 (4.7%)
> 20/200–20/100	117 (6.0%)	108 (7.2%)	225 (6.6%)
> 20/100 – 20/50	162 (8.4%)	168 (11.2%)	330 (9.6%)
20/40	56 (2.9%)	68 (4.5%)	124 (3.6%)
20/33	94 (4.9%)	116 (7.7%)	210 (6.1%)
20/25	151 (7.8%)	280 (18.7%)	431 (12.5%)
20/20 or better	1296 (66.9%)	660 (44.1%)	1956 (56.9%)
**Biometric measures: mean (SD)**			
Axial length (mm)	23.6 (1.3)	23.7 (1.3)	23.6 (1.3)
Corneal curvature radius (mm)	7.83 (0.25)	7.84 (0.26)	7.84 (0.26)
Axial length/corneal curvature radius ratio	3.01 (0.15)	3.03 (0.16)	3.02 (0.15)
Anterior chamber depth (mm)	3.62 (0.3)	3.57 (0.3)	3.60 (0.3)

*SER* spherical equivalent, *SD* standard deviation, *SD* standard deviation.

*Defined as cycloplegic spherical equivalent ≤ − 0.5 D in either eye.

**Defined as cycloplegic spherical equivalent ≤ − 6.0 D in either eye.

### Correlations of cycloplegic refractive error with biometric measures and UCVA

**Figure 1:**
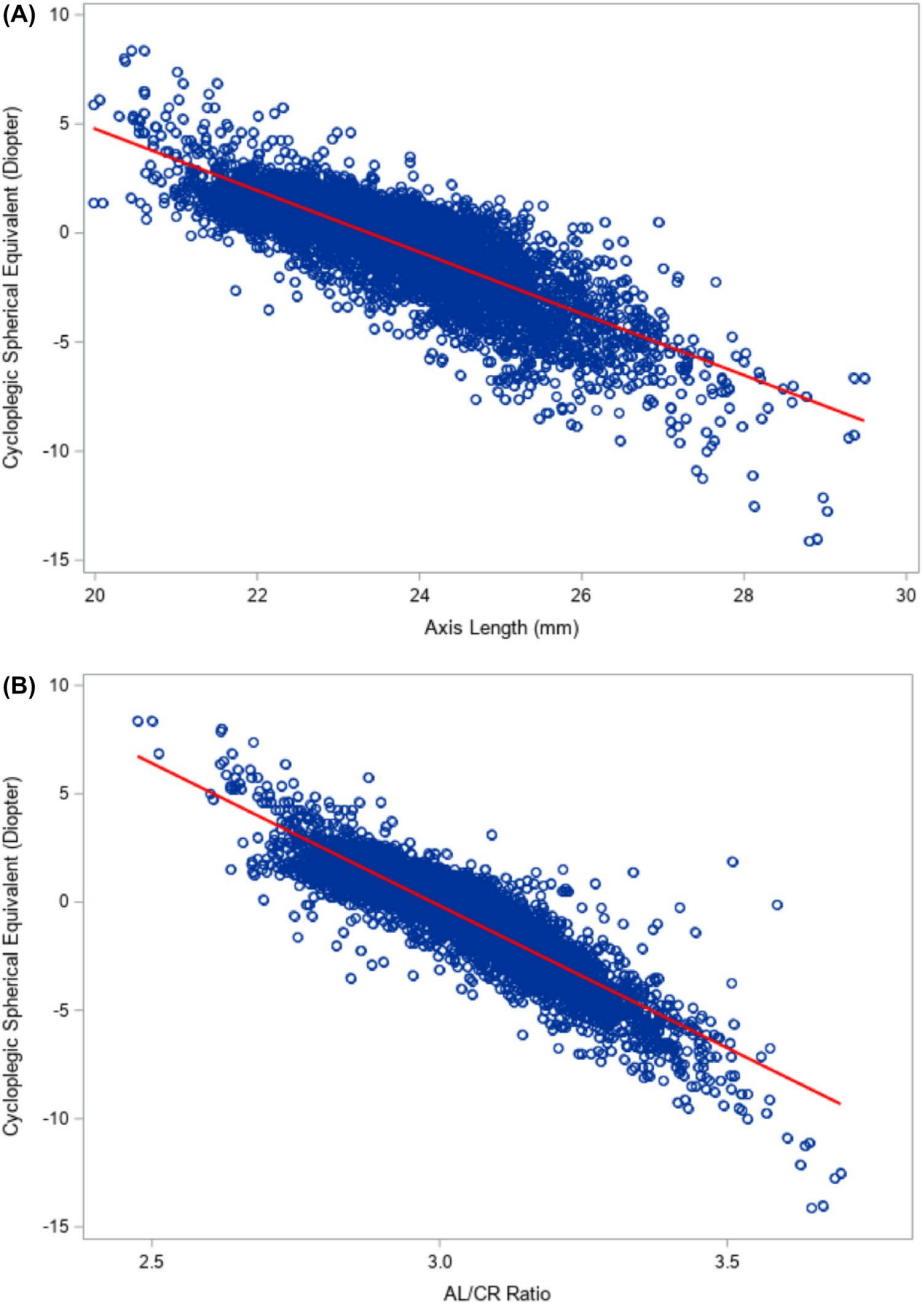
Scatterplots for cycloplegic spherical equivalent vs. Axial length (**A**) and AL/CR ratio (**B**). The red line is the linear regression line from the equation: spherical equivalent = 33.0 − 1.41 × Axial length in (**A**); The linear regression line is from the equation: spherical equivalent = 39.3 − 13.1 × AL/CR ratio in (**B**).

Among all students, cycloplegic spherical equivalent was significantly correlated with axial length (r = − 0.82, p < 0.0001, Fig. 1A), AL/CR ratio (r = − 0.90, p < 0.0001, Fig. 1B), anterior chamber depth (r = − 0.46, p < 0.0001), and UCVA (r = 0.79, p < 0.0001), However, cycloplegic spherical equivalent was not significantly correlated with corneal curvature radius (r = 0.02, p = 0.15, Table 2).

**Table 2:** Correlation of cycloplegic spherical equivalent with ocular biometric measures and uncorrected visual acuity.

Measure	Correlation with cycloplegic refractive error	
	Pearson correlation coefficient	p-value
Axial length (AL)	− 0.82	< 0.001
Corneal curvature radius (CR)	0.02	0.15
AL/CR ratio	− 0.90	< 0.001
Anterior chamber depth	− 0.46	< 0.001
Uncorrected visual acuity	0.79	< 0.001

When these correlations were assessed by the age of students, the correlation of cycloplegic spherical equivalent with AL and AL/CR ratio was found to increase with age (Table 3). The correlation coefficient increased from − 0.48 for 5–6-year-olds to − 0.81 for 17-year-olds for AL, and it increased from − 0.61 for 5–6-year-olds to − 0.91 for 17-year-olds for AL/CR ratio (Table 3).

**Table 3:** Correlation of cycloplegic spherical equivalent with axial length and AL/CR ratio for each age group.

Age (years)	# of students	Pearson correlation coefficient*	
		Axial length	AL/CR ratio
5–6	714	− 0.48	− 0.61
7	586	− 0.55	− 0.72
8	428	− 0.63	− 0.77
9	299	− 0.62	− 0.80
10	202	− 0.66	− 0.80
11	239	− 0.73	− 0.85
12	191	− 0.69	− 0.83
13	165	− 0.68	− 0.86
14	126	− 0.69	− 0.90
15	121	− 0.79	− 0.86
16	131	− 0.76	− 0.91
17	119	− 0.81	− 0.91
18	115	− 0.76	− 0.90

*AL/CR ratio* axial length/corneal curvature radius ratio.

*All the Pearson Spearman correlation coefficients are statistically significant.

### Association of AL/CR ratio and myopia

**Figure 2:**
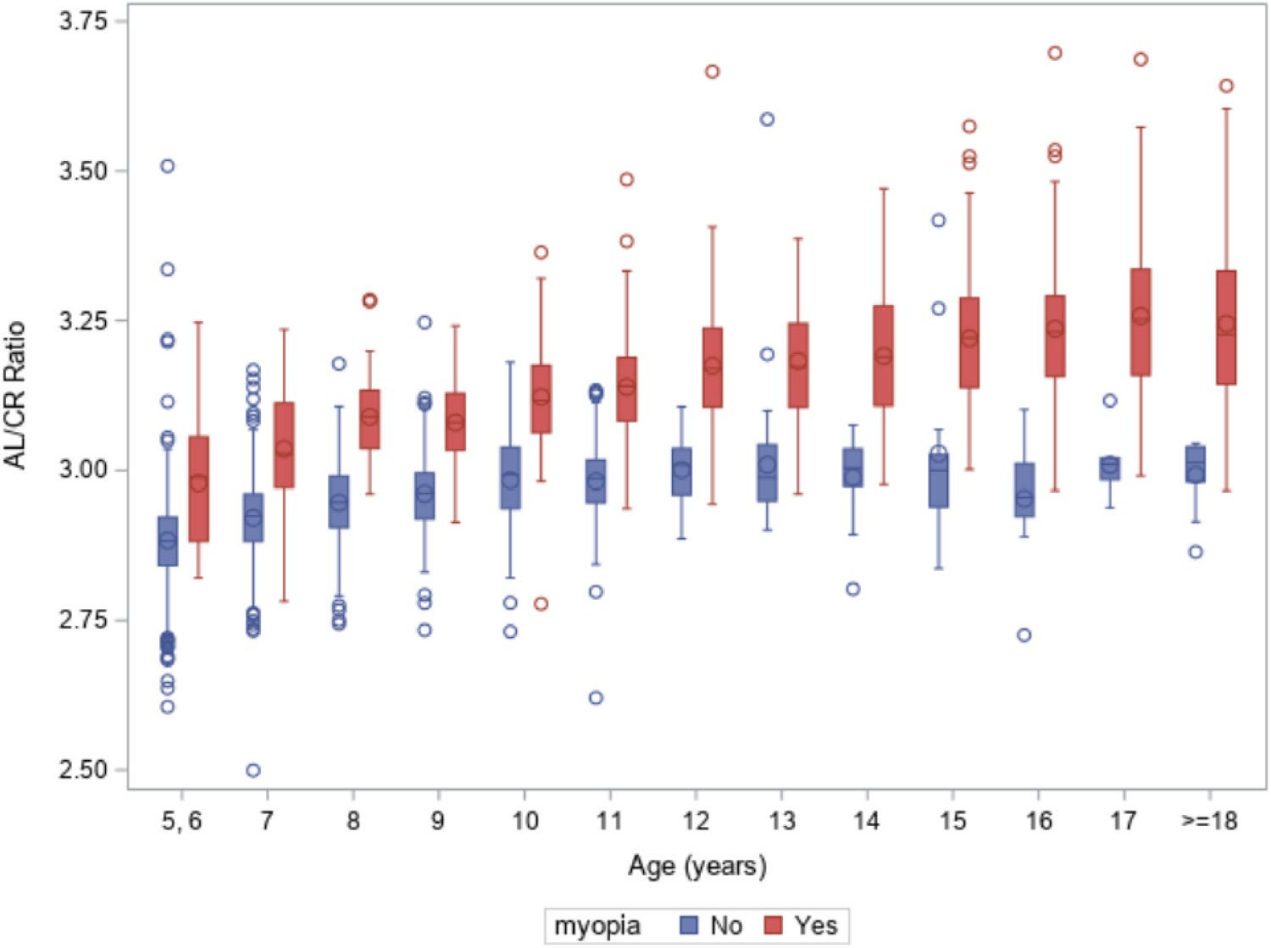
Boxplots for distribution of AL/CR by myopia status and age group of students.

Overall, the mean of AL/CR was significantly larger in students with myopia than students without myopia (3.17 vs. 2.93, p < 0.0001, Table 4). These significant differences remained in each age group of students (all p < 0.0001), although the AL/CR ratio tended to be larger in older students than younger students for both students without myopia and students with myopia (Fig. 2).

**Table 4:** Comparison of AL/CR ratio between students with and without myopia.

Age (years)	Students without myopia		Students without myopia		p-value
	n	Mean (SD)	n	Mean (SD)	
5–6	700	2.88 (0.07)	14	2.98 (0.12)	< 0.0001
7	542	2.92 (0.07)	44	3.04 (0.10)	< 0.0001
8	344	2.95 (0.07)	84	3.09 (0.06)	< 0.0001
9	192	2.96 (0.07)	107	3.08 (0.07)	< 0.0001
10	124	2.98 (0.07)	78	3.12 (0.09)	< 0.0001
11	104	2.98 (0.07)	135	3.14 (0.09)	< 0.0001
12	48	3.00 (0.05)	143	3.17 (0.10)	< 0.0001
13	46	3.01 (0.11)	119	3.18 (0.10)	< 0.0001
14	22	2.99 (0.07)	104	3.19 (0.12)	< 0.0001
15	13	3.03 (0.16)	108	3.22 (0.12)	< 0.0001
16	9	2.95 (0.11)	122	3.24 (0.13)	< 0.0001
17	10	3.01 (0.05)	109	3.26 (0.13)	< 0.0001
18	13	2.99 (0.06)	102	3.24 (0.14)	< 0.0001
All age groups combined	2167	2.93 (0.08)	1269	3.17 (0.12)	< 0.0001

*AL/CR ratio* axial length/corneal curvature radius ratio, *SD* standard deviation.

### Area under ROC curve (AUC) for myopia detection using biometric measures and UCVA

**Figure 3:**
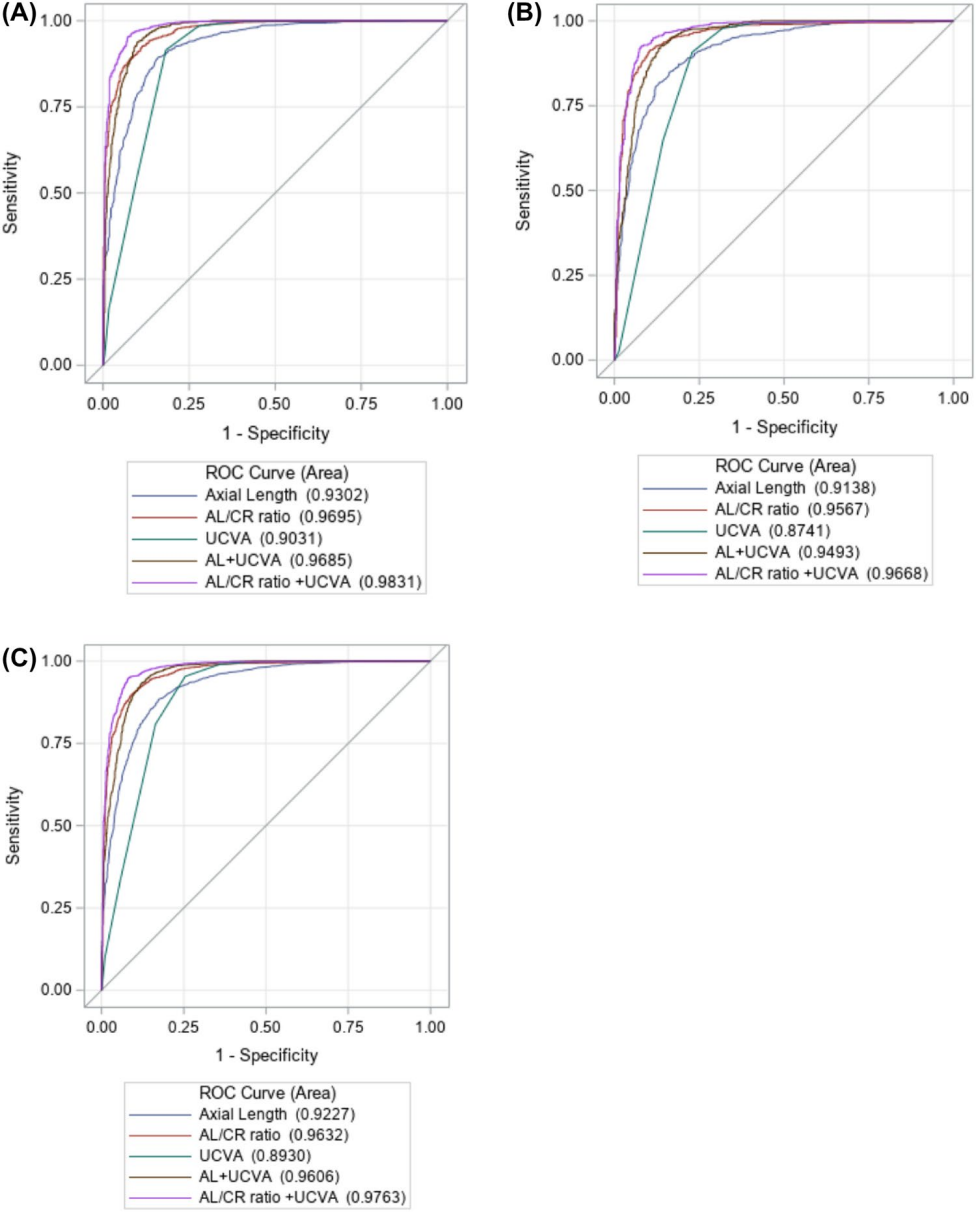
ROC curves using various measures for predicting myopia in development data (**A**), validation data (**B**) and combined data (**C**) using ocular biometric measures, UCVA and their combinations.

The AUC and its 95% CI for the detection of myopia using individual biometric measures and in combination with UCVA are shown in Table 5. AL/CR ratio had a higher AUC (0.963, 95% CI 0.957–0.969) than AL (AUC = 0.922, 95% CI 0.912–0.931) and UCVA (AUC = 0.893, 95% CI 0.881–0.906). The combination of AL/CR and UCVA improved the AUC to 0.976 (95% CI 0.971–0.981), which was significantly higher than that from the combination of AL and UCVA (AUC = 0.960, 95% CI 0.954–0.967) but was similar to the AUC from the combination of AL, AL/CR and UCVA (AUC = 0.979, 95% CI 0.974–0.984 Fig. 3). Similar AUC results were seen in the development dataset and validation dataset (Table 5, Fig. 3).

**Table 5:** Area under ROC curve for detecting myopia using single biometric measure or uncorrected visual acuity or their combinations.

Measurement	AUC (95% CI)		
	Development dataset	Validation dataset	Combined dataset
AL	0.930 (0.918, 0.942)	0.911 (0.897, 0.926)	0.922 (0.912, 0.931)
AL/CR ratio	0.970 (0.963, 0.977)	0.956 (0.946, 0.967)	0.963 (0.957, 0.969)
UCVA	0.903 (0.886, 0.920)	0.874 (0.854, 0.894)	0.893 (0.881, 0.906)
AL + UCVA	0.969 (0.960, 0.977)	0.949 (0.937, 0.960)	0.960 (0.954, 0.967)
AL/CR ratio + UCVA	0.983 (0.978, 0.989)	0.967 (0.957, 0.976)	0.976 (0.971, 0.981)
AL + AL/CR ratio + UCVA	0.986 (0.980, 0.991)	0.970 (0.961, 0.978)	0.979 (0.974, 0.984)

*AL* axial length, *AL/CR ratio* axial length/corneal curvature radius ratio, *UCVA* uncorrected visual acuity, *AUC* area under ROC curve, *95% CI* 95% confidence interval.

### Sensitivity and specificity of myopia detection using individual biometric measures and UCVA

The optimal age-specific cutoff values of individual biometric measure (AL and AL/CR ratio) that yielded high sensitivity and specificity is shown in Table 6. Using age-specific AL cutoff value (23.5 mm for age < 10 years, 24.0 mm for age 10–14 years, and 24.2 mm for age ≥ 15 years) for detecting myopia yielded sensitivity of 81.6% (95% CI 78.6–84.6%) and specificity of 83.2% (95% CI 81.2–85.2%) in the development dataset, and slightly lower sensitivity (80.8%, 95% CI 77.7–84.0%) and specificity (81.1%, 95% CI 78.5–83.3%) in the validation dataset.

**Table 6:** Sensitivity and specificity for detecting myopia using single ocular biometrics or uncorrected visual acuity in the development dataset and validation dataset.

	Measure	Development dataset		Validation dataset		Combined dataset	
	Cutoff	Sensitivity (%)	Specificity (%)	Sensitivity (%)	Specificity (%)	Sensitivity (%)	Specificity (%)
**Age (years)**							
< 10	> 23.5	77.7	84.5	69.7	82.3	72.7	83.8
10–14	> 24.0	79.6	75.9	82.3	71.7	80.8	74.3
≥ 15	> 24.2	85.9	92.0	87.4	84.2	86.6	88.6
**All ages**		81.6	83.2	80.8	81.1	81.2	82.4
	**AL/CR ratio**						
< 10	> 3.00	85.1	88.2	80.7	89.4	82.3	88.7
10–14	> 3.06	84.9	84.7	87.4	89.8	86.0	86.6
≥ 15	> 3.08	90.6	96.0	89.4	84.2	90.0	90.9
**All ages**		87.0	87.8	86.4	89.4	86.7	88.4
	**UCVA**						
**All ages**	< 20/25	72.2	98.4	77.1	90.7	74.6	95.3
**All ages**	< 20/32	60.4	99.8	68.3	97.7	64.3	98.9
**All ages**	< 20/40	52.2	99.9	59.4	99.2	55.7	99.6

*AL* axial length, *AL/CR ratio* axial length/corneal curvature radius ratio, *UCVA* uncorrected visual acuity.

Using age-specific cutoff value of AL/CR ratio (3.00 for age < 10 years, 3.06 for age 10–14 years, and 3.08 for age ≥ 15 years) yielded higher sensitivity and specificity than AL in both the development dataset (sensitivity 87.0%, 95% CI 84.4–90.0%, specificity 87.8%, 95% CI 86.0–89.6%) and the validation dataset (sensitivity 86.4%, 95% CI 83.7–89.1%, specificity 89.4%, 95% CI 87.3–91.4%).

Among students less than 10 years old, using AL/CR ratio greater than 3.00 as myopia positive yielded sensitivity 85.1% (95% CI 77.9–92.3%), specificity 88.2% (95% CI 86.3–90.2%) in the training dataset, sensitivity 80.7% (95% CI 74.4–86.9%) and specificity 89.4% (95% CI 87.2–91.6%) in validation dataset. Among students aged 10 to 14 years old, using AL/CR ratio greater than 3.06 as myopia positive yielded 84.9% (95% CI 81.0–88.8%) in sensitivity, 84.7% (95% CI 79.9–89.5%) in specificity in the training dataset, 87.4% (95% CI 83.3–91.5%) in sensitivity and 89.8% (84.5–95.0%) in specificity in the validation dataset. Among students aged 15 years or older, using AL/CR ratio greater than 3.08 as myopia positive yielded very high sensitivity (90.6%, 95% CI 86.9–94.3%) and specificity (96.0%, 95% CI 88.3–100%) in the training dataset, and lower sensitivity (89.4%, 95% CI 85.2–93.6%) and specificity (84.2%, 95% CI 67.8–100%) in the validation dataset.

The use of UCVA for detecting myopia provided lower sensitivity yet higher specificity than AL and AL/CR. Specifically, UCVA worse than 20/25 provided sensitivity of 72.2% (95% CI 68.6–75.7%) and specificity of 98.4% (95% CI 97.5–99.0%) in the development dataset, and sensitivity of 77.1% (95% CI 73.5–80.3%) and specificity of 90.7% (95% CI 88.6–92.5%) in the validation dataset.

### Sensitivity and specificity of myopia detection using combination of AL/CR and UCVA

The sensitivity and specificity of using the combination of AL/CR and UCVA are shown in Table 7. Using AL/CR larger than age-specific cutoff value (3.00 for age < 10 years, 3.06 for age 10–14 years, and 3.08 for age ≥ 15 years) or UCVA worse than age-specific cutoff value (20/32 for age < 10 years, 20/25 for age ≥ 10 years) as myopia positive, the sensitivity and specificity were 91.6% (95% CI 89.4–93.7%) and 87.5% (95% CI 85.7–89.3%) respectively in the development dataset. These cutoff values for the combination of AL/CR and UCVA were well validated with sensitivity of 92.2% (95% CI 90.1–94.3%) and specificity of 86.3% (95% CI 84.0–88.6%) in the validation dataset. The sensitivity and specificity from combination of AL/CR and UCVA for each age group were also reported in Table 7. In the training dataset, the age group of 15 years or older had the highest sensitivity (> 92%) and specificity (> 92%), and the sensitivity remained very high in validation dataset (> 90%).

**Table 7:** Sensitivity and specificity for detecting myopia using combinations of AL/CR ratio and UCVA in the development dataset and validation dataset.

Age (years)	Cutoff		Development dataset		Validation dataset		Combined dataset	
	AL/CR	UCVA	Sensitivity (%)	Specificity (%)	Sensitivity (%)	Specificity (%)	Sensitivity (%)	Specificity (%)
< 10	> 3.00	< 20/32	88.3	88.1	85.8	87.0	86.8	87.7
10–14	> 3.06	< 20/32	87.4	83.8	90.9	89.0	88.9	85.7
≥ 15	> 3.08	< 20/32	92.3	96.0	93.2	84.2	92.7	90.9
**All ages**			89.3	87.6	90.4	87.2	89.8	87.4
< 10	> 3.00	< 20/25	91.5	86.8	89.0	80.7	90.0	84.3
10–14	> 3.06	< 20/25	90.4	83.8	93.3	83.5	91.7	83.7
≥ 15	> 3.08	< 20/25	94.4	92.0	95.7	79.0	95.0	86.4
**All ages**			92.0	86.4	93.0	81.0	92.5	84.2
< 10	> 3.00	< 20/32	88.3	88.1	85.8	87.0	86.8	87.7
10–14	> 3.06	< 20/25	90.4	83.8	93.3	83.5	91.7	83.7
≥ 15	> 3.08	< 20/25	94.4	92.0	95.7	79.0	95.0	86.4
**All ages**			91.6	87.5	92.2	86.3	91.9	87.0

*AL* axial length, *AL/CR ratio* axial length/corneal curvature radius ratio, *UCVA* uncorrected visual acuity.

In the combined dataset, the combination of AL/CR and UCVA yielded sensitivity of 91.9% (95% CI 90.4–93.4%) specificity of 87.0% (95% CI 85.6–88.4%), positive predictive value of 80.6% (95% CI 78.5–82.6%) and negative value of 94.8% (95% CI 93.8–95.8%) (Table 7).

## Discussion

In this large cross-sectional school-based study, we evaluated the performance of ocular biometric measures and UCVA for detecting myopia among Chinese school students. We found that AL/CR ratio was highly correlated with cycloplegic refractive error and that age-specific AL/CR cutoff values (3.00 for age < 10 years, 3.06 for age 10–14 years, and 3.08 for age ≥ 15 years) detected myopia with high sensitivity (87%) and specificity (88%). Combining AL/CR with age-specific UCVA (< 20/32 for age < 10 years, and 20/25 for age ≥ 10 years) improved the sensitivity (92%) and slightly decreased specificity (87%). Our findings support that AL/CR ratio alone or in combination with UCVA can be used as an objective tool to screen for myopia or estimate myopia prevalence in large epidemiological studies with limited resources for cycloplegic refraction.

A previous study of 3922 Chinese school children (aged 6–12 years old) by He et al.^12^ showed that using an AL/CR ratio of 3.00 as the cutoff value provided a sensitivity of 83% and a specificity of 82%, and combining the AL/CR with UCVA (i.e., myopia positive if AL/CR > 2.95 AND UCVA 20/25 or worse) improved the specificity to 91% with sensitivity remaining at 83%. Instead of using the same cutoff value of Al/CR ratio regardless of age as in He’s study, our study applied age-specific AL/CR optimal cutoff value that provided higher sensitivity (87%) and specificity (88%). The optimal age-specific cutoff point in this study was developed in the development dataset from students in Jinyun and independently validated in the validation dataset from students in Hangzhou. The combination of age-specific AL/CR and UCVA in this study also provided much higher sensitivity (91.9% vs. 83.0%) than the study of He et al. yet slightly lower specificity (87.6% vs. 91.0%)^12^. The differences in sensitivity and specificity between these two studies could be due to the differences in the cutoff value of AL/CR ratio, the study characteristics of school children such as age, refractive error status and the device for biometric measures (IOL Master in study of He et al. and NIDEK Optical Biometer AL-scan in this study). In spite of these differences, both studies found that AL/CR was better than AL and UCVA for detecting myopia and that combining AL/CR with UCVA improved the sensitivity, supporting the use of AL/CR for myopia detection.

Consistent with previous studies^9–15^, our study also found that AL/CR ratio was more correlated (r = − 0.90) with cycloplegic spherical equivalent than AL (r = − 0.82) overall and across each age from 5 to 18 years old, and their correlation with cycloplegic spherical equivalent increased with age. However, the correlation of AL/CR ratio with cycloplegic spherical equivalent in this study was higher than those in previous studies that reported correlation coefficients ranging from − 0.78 to − 0.89 in young adults and ranging from − 0.61 to − 0.78 in school children^10–16^.

To find the optimal cutoff values of AL/CR for myopia detection, this study investigated all possible age-specific cutoff values of AL/CR ratio and its various combinations with UCVA in the development dataset and then applied the selected optimal cutoff values to the validation dataset for independent validation. We found that the age-specific optimal cutoff value (3.00 for age < 10 years, 3.06 for age 10–14 years, and 3.08 for age ≥ 15 years) in AL/CR ratio provided high sensitivity (87%) and specificity (88%) in the development dataset and was well validated in the validation dataset (sensitivity 86% with specificity 89%). Our optimal cutoff value was age-specific, which is different from the single cutoff value of 3.00 used by the He et al. that yielded lower sensitivity of 83% and specificity of 82%^12^.

As UCVA is easy to measure, we evaluated whether combining UCVA with AL/CR ratio improved myopia detection. Although we explored age-specific cutoff values of AL/CR ratio in combinations with various age-specific UCVA cutoff values and applied logic combinations of “AND or “OR”, we found that the best combination of age-specific cutoff value of AL/CR ratio (3.00 for age < 10 years, 3.06 for age 10–14 years, and 3.08 for age ≥ 15 years) and age-specific cutoff value of UCVA (< 20/32 for age < 10 years, and 20/25 for age ≥ 10 years) increased the sensitivity in both the development dataset (91.6%) and the validation dataset (92.2%) while retaining similar specificity (87.5% in the development dataset and 86.3% in the validation dataset). The study of He et al.^12^ also explored the combination of AL/CR ratio and UCVA and found that the optimal combination (defined myopia positive as AL/CR ratio > 2.95 and UCVA < 20/25) yielded a lower sensitivity (83.0%) than our study but a similar specificity (90.6%).

Unlike refractive error, ocular biometric measures are not affected by the accommodation, so administering cycloplegic eyedrops is not needed for measuring ocular biometrics, which can be obtained from modern biometers quickly, objectively, and reliably under noncycloplegic conditions. Therefore, biometric measures overcome the challenges of administering cycloplegic eyedrops in young children or in large-scale studies. Our study demonstrates that AL/CR ratio alone or in combination with UCVA can detect myopia with high sensitivity and specificity, thus providing a convenient tool for identifying children with a high likelihood of myopia that requires cycloplegic refractive measurement to confirm or for estimating the prevalence rate of myopia in large epidemiological studies when the resources for cycloplegic refraction are limited.

Using the same data from this study, we previously developed and validated a prediction model for predicting cycloplegic refractive error based on the noncycloplegic refractive error from a NIDEK autorefractor, demographics, BCVA, and ocular biometric measures^21^. Applying the predicted cycloplegic refractive error from the prediction model yielded a good sensitivity (85%) and an excellent specificity (98%), and the combination of predicted cycloplegic refractive error and UCVA improved the detection of myopia with a sensitivity of 92% and a specificity of 93%, which are similar to the sensitivity of this study (92%) but higher specificity (87%) than this study that only considered the AL/CR and UCVA. He et al. also recently developed a prediction model based on the percentiles of AL, AL/CR ratio and age that yielded a sensitivity of 87.4% and a specificity of 88.2% among children of 5–18 years old^16^. The simple application of AL/CR ratio alone or in combination with UCVA or these more complicated prediction models that considered other measures (e.g. age, noncycloplegic refractive error, biometric measures) could potentially be used to determine the myopia risk or to estimate the myopia prevalence in epidemiological studies in which administering cycloplegic agent to all participants is not feasible.

The strength of this study is the large sample size and the standard study protocol for the biometric measures, UCVA and cycloplegic refractive error. Unlike other studies, this study explored all possible age-specific cutoff values of AL/CR ratio and its combination with UCVA in a development dataset to find the age-specific optimal cutoff value of the AL/CR ratio and applied the selected optimal cutoff value to a validation dataset for independent validation. However, this study is limited in that 0.5% tropicamide was used as the cycloplegic agent instead of 1% tropicamide or other more powerful cycloplegic agent. In this study, we instilled one drop of 0.5% tropicamide in each eye, followed by the second, third and fourth drop of 0.5% tropicamide every 5 min. Thirty minutes after the fourth drop of 0.5% tropicamide was instilled, cycloplegic refractive error measurements were then taken from each eye. This procedure allows approximately 45 min to achieve maximum cycloplegic effect. In a previous paper^20^ from this same myopia study, we reported that 0.5% tropicamide yielded cycloplegic effect of 0.92 D, which was within the range of the cycloplegic effect of 0.60–1.23 D reported in other studies that used different type of cycloplegic agents (e.g., 1% atropine sulfate, 1% cyclopentolate etc.)^23–26^. In spite of these, it is still possible that the full cycloplegic refraction might not be achieved from using 0.5% tropicamide.

In conclusion, this study evaluated the performance of using ocular biometric measures, particularly AL/CR ratio and its combination with UCVA, for detecting myopia in Chinese school students. We identified and validated the age-specific optimal cutoff value of AL/CR ratio that provided high sensitivity and specificity. We found that combining UCVA with AL/CR ratio improved the sensitivity for detecting myopia. While cycloplegic refraction, the gold standard for evaluating myopia, should be used in the clinical care of myopia, the AL/CR ratio alone or in combination with UCVA may provide a useful tool for screening myopia or estimating myopia prevalence in epidemiological studies in which administering a cycloplegic agent to all participants is not feasible.

## Data Availability

The datasets nalysed during the current study are available from the corresponding authors upon reasonable request.

## References

[1] Sankaridurg P, Tahhan N, Kandel H (2021). IMI impact of myopia. Investig. Ophthalmol. Vis. Sci..

[2] Morgan IG, Wu PC, Ostrin LA (2021). IMI Risk factors for myopia. Investig. Ophthalmol. Vis. Sci..

[3] Cho BJ, Shin JY, Yu HG (2016). Complications of pathologic myopia. Eye Contact Lens.

[4] Wolffsohn JS, Flitcroft DI, Gifford KL (2019). IMI—Myopia control reports overview and introduction. Investig. Ophthalmol. Vis. Sci..

[5] Flitcroft DI, He M, Jonas JB (2019). IMI—Defining and classifying myopia: A proposed set of standards for clinical and epidemiologic studies. Investig. Ophthalmol. Vis. Sci..

[6] Koh V, Tan C, Nah G (2014). Correlation of structural and electrophysiological changes in the retina of young high myopes. Ophthalmic Physiol. Opt..

[7] Wu HM, Seet B, Yap EP, Saw SM, Lim TH, Chia KS (2001). Does education explain ethnic differences in myopia prevalence? A population-based study of young adult males in Singapore. Optom. Vis. Sci..

[8] Yotsukura E, Torii H, Inokuchi M (2019). Current prevalence of myopia and association of myopia with environmental factors among school children in Japan. JAMA Ophthalmol..

[9] Ojaimi E, Rose KA, Morgan IG (2005). Distribution of ocular biometric parameters and refraction in a population-based study of Australian children. Investig. Ophthalmol. Vis. Sci..

[10] Kimura S, Hasebe S, Miyata M, Hamasaki I, Ohtsuki H (2007). Axial length measurement using partial coherence interferometry in myopic children: Repeatability of the measurement and comparison with refractive components. Jpn. J. Ophthalmol..

[11] Ip JM, Huynh SC, Kifley A (2007). Variation of the contribution from axial length and other oculometric parameters to refraction by age and ethnicity. Investig. Ophthalmol. Vis. Sci..

[12] He X, Zou H, Lu L (2015). Axial length/corneal radius ratio: Association with refractive state and role on myopia detection combined with visual acuity in Chinese school children. PLoS One.

[13] Grosvenor T, Scott R (1994). Role of the axial length/corneal radius ratio in determining the refractive state of the eye. Optom. Vis. Sci..

[14] Grosvenor T (1988). High axial length/corneal radius ratio as a risk factor in the development of myopia. Am. J. Optom. Physiol. Opt..

[15] Gonzalez Blanco F, Sanz Fernandez JC, Munoz Sanz MA (2008). Axial length, corneal radius, and age of myopia onset. Optom. Vis. Sci..

[16] He XG, Sankaridurg P, Naduvilath T (2021). Normative data and percentile curves for axial length and axial length/corneal curvature in Chinese children and adolescents aged 4–18 years. Br. J. Ophthalmol..

[17] Magome K, Morishige N, Ueno A, Matsui TA, Uchio E (2021). Prediction of cycloplegic refraction for noninvasive screening of children for refractive error. PLoS One.

[18] Sankaridurg P, He X, Naduvilath T (2017). Comparison of noncycloplegic and cycloplegic autorefraction in categorizing refractive error data in children. Acta Ophthalmol..

[19] Foo VH, Verkicharla PK, Ikram MK (2016). Axial length/corneal radius of curvature ratio and myopia in 3-year-old children. Transl. Vis. Sci. Technol..

[20] Gu F, Gao HM, Zheng X (2021). Effect of cycloplegia on refractive error measure in Chinese school students. Ophthal. Epidemiol..

[21] Wang JY, Wang XY, Gao HM (2022). Prediction for cycloplegic refractive error in Chinese school students: model development and validation. Transl. Vis. Sci. Technol..

[22] Youden WJ (1950). Index for rating diagnostic tests. Biometrics.

[23] Fotedar R, Rochtchina E, Morgan I, Wang JJ, Mitchell P, Rose KA (2007). Necessity of cycloplegia for assessing refractive error in 12-year-old children: A population-based study. Am. J. Ophthalmol..

[24] Hu YY, Wu JF, Lu TL (2015). Effect of cycloplegia on the refractive status of children: The Shandong children eye study. PLoS One.

[25] Zhao J, Mao J, Luo R, Li F, Pokharel GP, Ellwein LB (2004). Accuracy of noncycloplegic autorefraction in school-age children in China. Optom. Vis. Sci..

[26] Liu X, Ye L, Chen C, Chen M, Wen S, Mao X (2020). Evaluation of the necessity for cycloplegia during refraction of Chinese children between 4 and 10 years old. J. Pediatr. Ophthalmol. Strabismus.

