# Author Correction: Ocular biometrics and uncorrected visual acuity for detecting myopia in Chinese school students

**DOI:** 10.1038/s41598-022-25893-w

**Published:** 2022-12-07

**Authors:** Ethan Zhao, Xinyi Wang, Huiyan Zhang, Eric Zhao, Jianyong Wang, Ying Yang, Fang Gu, Lei Gu, Jianyao Huang, Ronghua Zhang, Gui-shuang Ying, Hongguang Cui

**Affiliations:** 1 Weill Cornell Medicine, New York, NY USA; 2 National School of Development, Peking University, Beijing, People’s Republic of China; 3 Hangzhou Vocational and Technical College, Hangzhou, People’s Republic of China; 4 School of Engineering and Applied Science, University of Pennsylvania, Philadelphia, PA USA; 5 Department of Ophthalmology, The First Affiliated Hospital, College of Medicine, Zhejiang University, Hangzhou, People’s Republic of China; 6 Center for Disease Control and Prevention of Jinyun County, Jinyun, Zhejiang People’s Republic of China; 7 Zhejiang Provincial Center for Disease Control and Prevention of Hangzhou, Hangzhou, People’s Republic of China; 8 Department of Ophthalmology, Central Hospital of Jinyun County, Jinyun, Zhejiang People’s Republic of China; 9 Center for Preventive Ophthalmology and Biostatistics, Department of Ophthalmology, Perelman School of Medicine, University of Pennsylvania, 3711 Market Street, Suite 801, Philadelphia, PA 19104 USA

Correction to: *Scientific Reports* https://doi.org/10.1038/s41598-022-23409-0, published online 04 November 2022

In the original version of this Article author Hongguang Cui was incorrectly assigned affiliation 4: “School of Engineering and Applied Science, University of Pennsylvania, Philadelphia, PA, USA”, and affiliation 10: “The First Affiliated Hospital, College of Medicine, Zhejiang University, 79 Qingchun Road, Hangzhou 310003, People’s Republic of China”, instead of affiliation 5. The correct affiliation 5 is listed below.

“Department of Ophthalmology, The First Affiliated Hospital, College of Medicine, Zhejiang University, Hangzhou, People’s Republic of China.”

Additionally, affiliation 10 was removed from this Article as it was a duplicate of affiliation 5.

The original Article has been corrected.

